# Supplementary figures and images for: Alpha-1 antitrypsin limits neutrophil extracellular trap disruption of airway epithelial barrier function
Source: Front Immunol. 2023 Jan 10;13:1023553. doi: 10.3389/fimmu.2022.1023553 (PMC9872031; doi:10.3389/fimmu.2022.1023553)

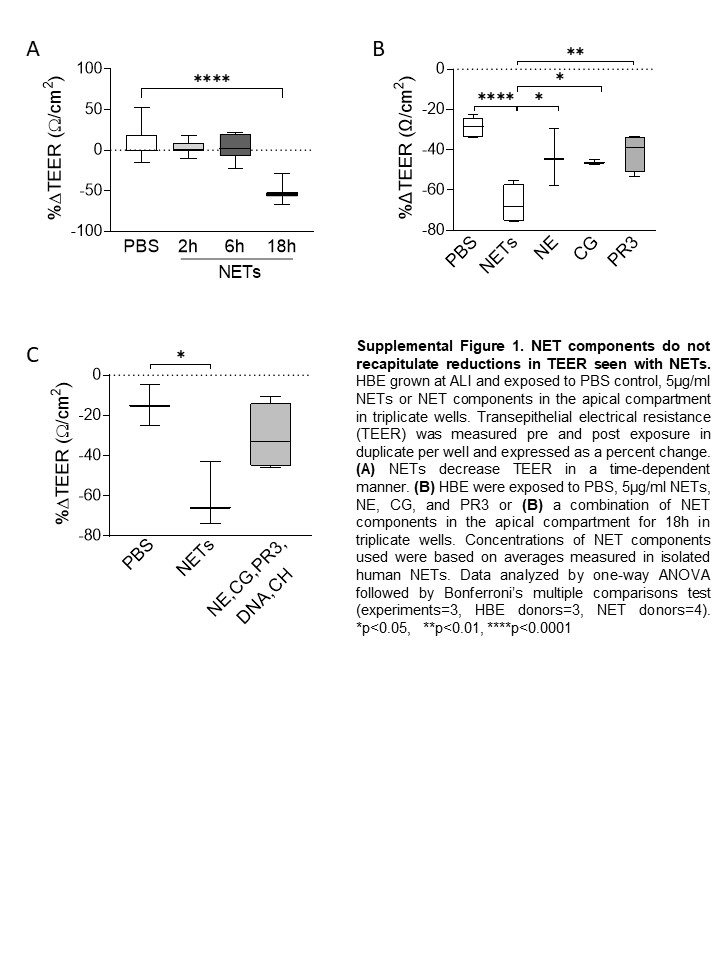

Supplement: Supplementary file 1 [file Image_1.jpg]

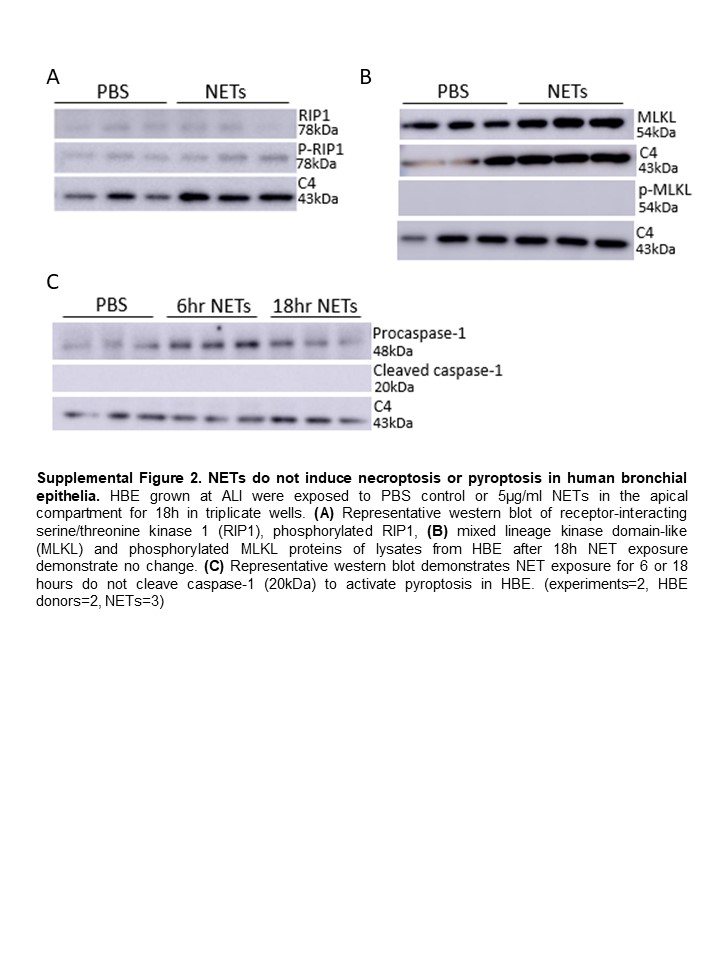

Supplement: Supplementary file 2 [file Image_2.jpg]

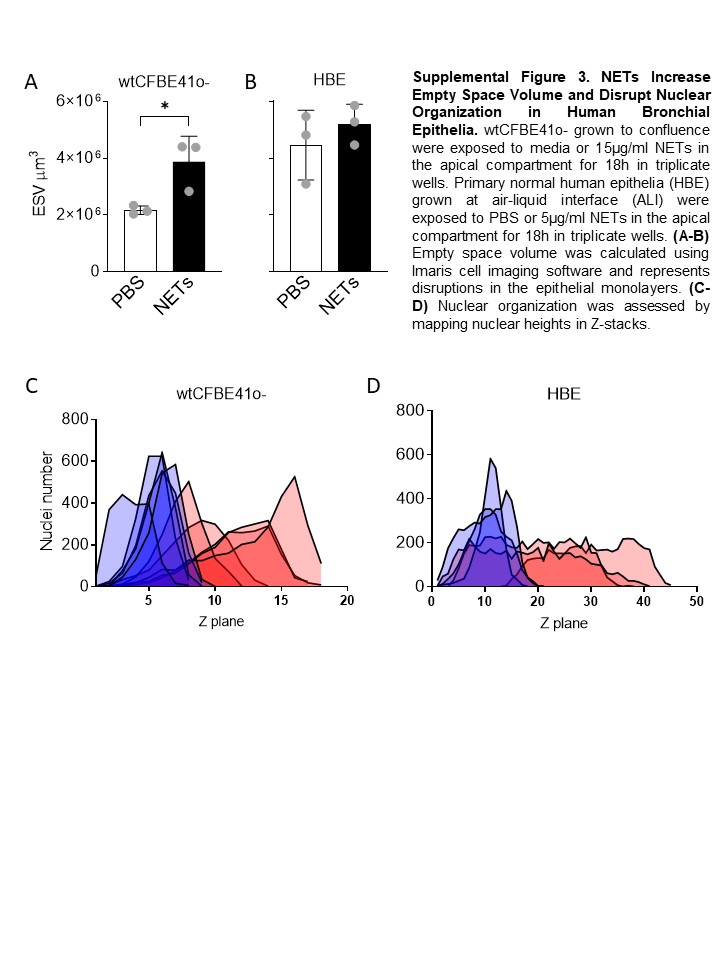

Supplement: Supplementary file 3 [file Image_3.jpg]

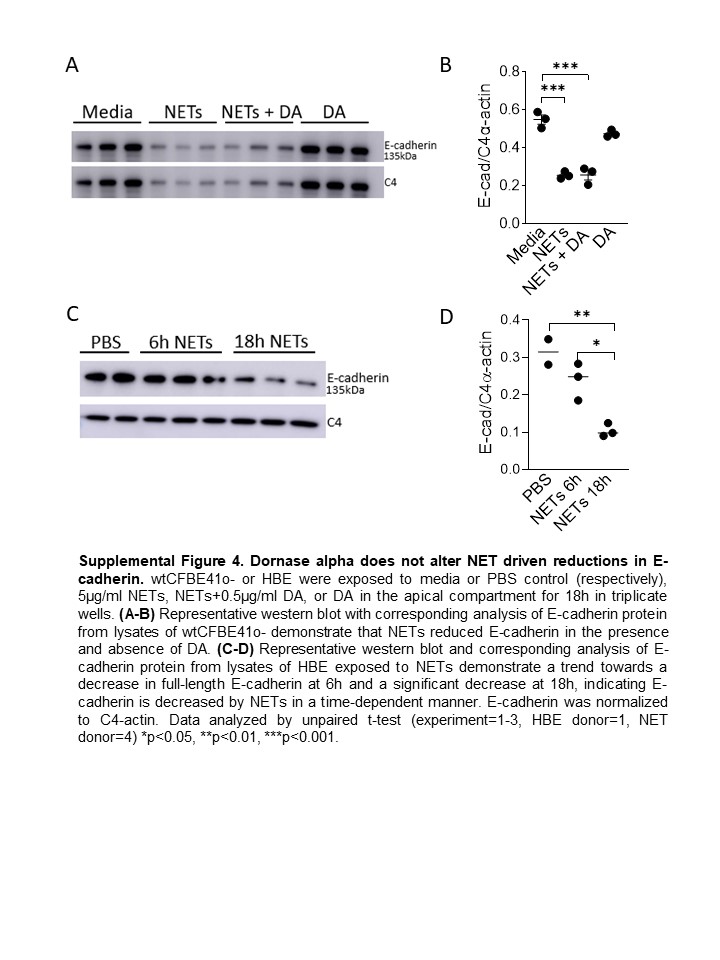

Supplement: Supplementary file 4 [file Image_4.jpg]

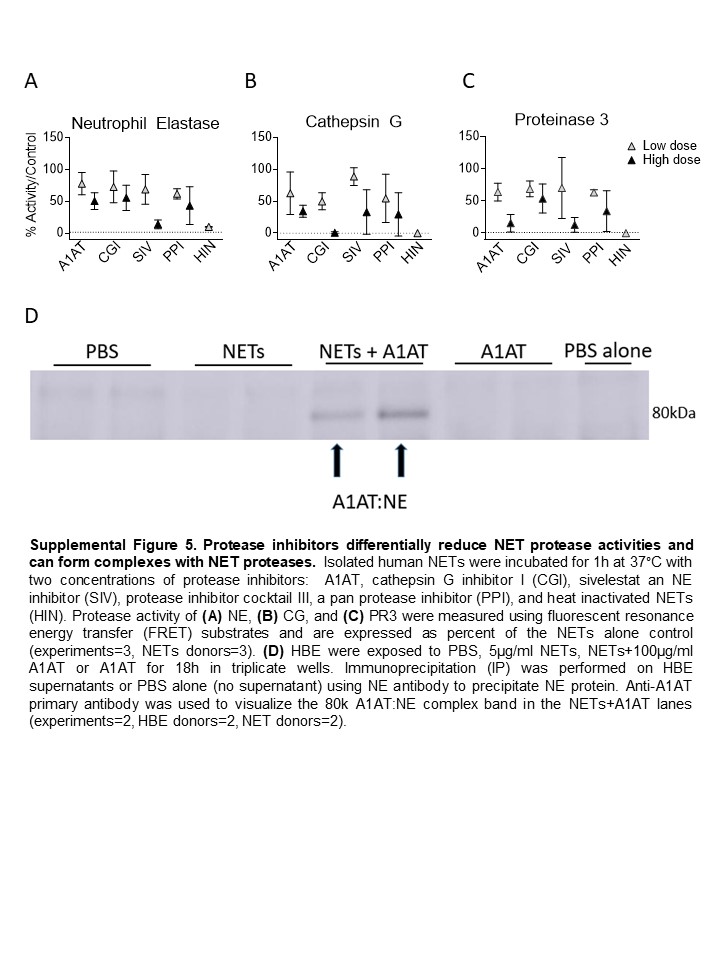

Supplement: Supplementary file 5 [file Image_5.jpg]

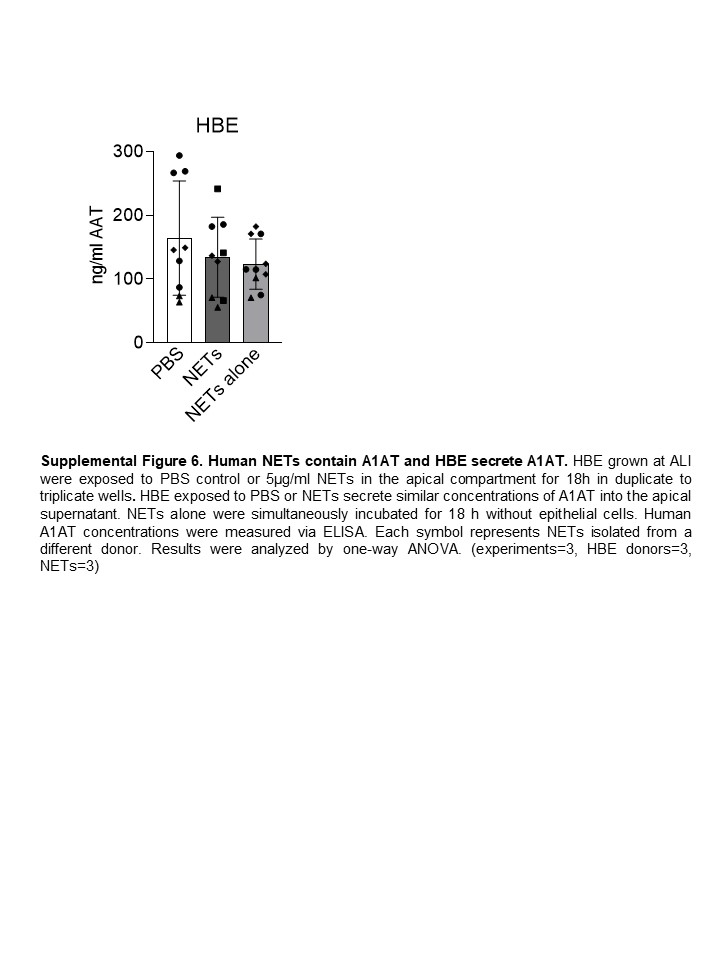

Supplement: Supplementary file 6 [file Image_6.jpg]
