# Supplementary material for: Alpha-1 antitrypsin limits neutrophil extracellular trap disruption of airway epithelial barrier function
Source: Front Immunol. 2023 Jan 10;13:1023553. doi: 10.3389/fimmu.2022.1023553 (PMC9872031; doi:10.3389/fimmu.2022.1023553)
Supplement: Supplementary file 7 [file Table_1.pdf]

Supplemental Table 1. NETs alter RNAs affecting apoptosis in human bronchial epithelia. List of differentially expressed genes associated with apoptosis found by RNA sequencing of HBE exposed to 5µg/ml NETs compared to PBS for 18h. Data analyzed using IPA (p=1.07e-13, experiments=3, HBE donors=3, NET donors=3).

Apoptosis					
Symbol	Fold Change	Symbol	Fold Change	Symbol	Fold Change
IL1RL1	104.7046	*HDAC9*	5.019643	*PBX1*	-2.89936
CD200	49.66355	*VAV1*	4.949888	*PTGS1*	-3.01138
MMP9	49.35035	*TIMP2*	4.81805	*LGR4*	-3.01154
SEMA7A	39.21162	*PHLDA2*	4.794256	*PTTG1*	-3.09511
CEACAM1	22.26556	*NOG*	4.709203	*WNT5A*	-3.24799
PLAUR	14.30711	*PIK3AP1*	4.528828	*WNT3A*	-3.59439
RASSF2	13.18982	*IL32*	4.515641	*FKBP5*	-3.85641
PLAU	10.91761	*TLR2*	4.487772	*PIK3R1*	-3.97341
MMP10	9.987298	*POLB*	4.475649	*ID1*	-3.97642
TIAM1	9.666262	*SOX9*	4.434872	*JAG2*	-4.05528
GPR132	8.60728	*CLCF1*	4.37477	*FGFR3*	-4.06072
GDF15	8.016345	*EREG*	4.200381	*ID3*	-4.20011
TREM1	7.543849	*CCND1*	3.948307	*VAV3*	-4.31285
IL1RN	7.038242	*MAP4K4*	3.876084	*ANGPT1*	-4.32759
ELOVL4	6.957872	*PTGS2*	3.831697	*HSD11B2*	-4.41233
DKK1	6.847217	*CEACAM5*	3.775852	*SDC2*	-4.5023
NT5E	6.753523	*TGFB1*	3.726483	*SNAI2*	-4.71535
GLIPR1	6.489568	*KRT17*	3.520094	*CAV1*	-5.04368
PHLDA1	6.403348	*RASD1*	3.487722	*TIMP3*	-5.35201
PLK3	6.377526	*PIK3IP1*	3.429813	*NGFR*	-5.55712
OSBP2	5.996882	*ITGA1*	3.152443	*MT3*	-6.51282
ADAM8	5.705564	*F3*	3.082455	*APLN*	-7.14273
FST	5.428863	*KIFC3*	2.944804	*GAS1*	-7.24106
EHD3	5.288924	*TXNRD1*	2.805253	*ID2*	-7.33517
SPHK1	5.132752	*SCD*	-2.69065	*TP63*	-7.46925
MMP1	5.041066	*PTHLH*	-2.78389	*DLL1*	-9.40422
CCL5	5.024486	*TRIM2*	-2.8052		
IRAK2	5.024247	*PDCD4*	-2.87253		
