# Supplementary material for: Alpha-1 antitrypsin limits neutrophil extracellular trap disruption of airway epithelial barrier function
Source: Front Immunol. 2023 Jan 10;13:1023553. doi: 10.3389/fimmu.2022.1023553 (PMC9872031; doi:10.3389/fimmu.2022.1023553)
Supplement: Supplementary file 8 [file Table_2.pdf]

Supplemental Table 2. NETs alter epithelial cell junction RNAs. List of functionally enriched RNAs for cellular components of anchored junctions and tight junctions found by RNA sequencing of HBE exposed to 5µg/ml NETs compared to PBS for 18h. Data analyzed using ToppGene (experiments=3, HBE donors=3, NET donors=3).

Cell Junction Organization	
Symbol	Fold Change
ACTB	2.89996469
KIFC3	2.944804134
CLDN4	3.207103512
TGFB1	3.726483192
MAP4K4	3.876084105
LAMB3	4.014288083
TLR2	4.487771729
ESAM	4.649265569
EPHB3	4.978859764
DKK1	6.847217229
LAMC2	8.371985395
TIAM1	9.666262173
ARHGAP22	-5.618308079
CAV1	-5.043682033
WNT4	-4.864411143
CLDN8	-4.838474606
SNAI2	-4.715354949
PIK3R1	-3.973412632
MTSS1	-3.870409255
WNT3A	-3.594393098
WNT5A	-3.247994985
